# Effect of environmental exposures on allergen sensitization and the development of childhood allergic diseases: A large-scale population-based study

**DOI:** 10.1016/j.waojou.2020.100495

**Published:** 2021-01-06

**Authors:** Chian-Feng Huang, Wei-Chu Chie, I-Jen Wang

**Affiliations:** a Institute of Epidemiology and Preventive Medicine, College of Public Health, National Taiwan University, Taipei 10055, Taiwan; b Miaoli General Hospital, Ministry of Health and Welfare, Miaoli 36054, Taiwan; c Department of Pediatrics, Taipei Hospital, Ministry of Health and Welfare, New Taipei City 24213, Taiwan; d School of Medicine, National Yang-Ming University, Taipei 112, Taiwan; e College of Public Health, China Medical University, Taichung 40402, Taiwan; f National Institute of Environmental Health Sciences, National Health Research Institutes, Miaoli 350, Taiwan; g National Taiwan University Hospital, National Taiwan University,Taipei 100, Taiwan

**Keywords:** Allergic diseases, Asthma, Atopic dermatitis, Allergen sensitization, Environmental exposures, OR, odd ratio, CI, confidence interval, AD, atopic dermatitis, AR, allergic rhinitis, ISAAC, International Study of Asthma and Allergies in Childhood, ETS, environmental tobacco smoke, SPTs, skin prick tests, HDMs, house dust mites

## Abstract

**Background:**

Changing environmental factors are likely responsible for the rising prevalence of allergic diseases in children. However, whether environmental exposures induce allergen sensitizations, and which allergen sensitization is related to the development of allergic diseases, is not clear. The study is aimed to investigate the association between environmental exposure, allergen sensitization, and the development of allergic diseases for further preventive intervention.

**Methods:**

We conducted the Taiwan Childhood Environment and Allergic diseases Study (TCEAS) in kindergarten children in Taiwan. Skin prick tests for 6 allergens were performed. Information on the development of allergic diseases and environmental exposure was collected using standardized questionnaires. Multiple logistic regressions were used to estimate the association between environmental factors, allergen sensitization, and the development of allergic diseases.

**Results:**

A total of 3192 children were recruited. 485 (15.2%) children had atopic dermatitis (AD), 1126 (35.3%) had allergic rhinitis (AR), and 552 (17.3%) had asthma. Children with environmental tobacco smoke exposure and fungi on the house wall had a higher risk of asthma, with ORs (95% CIs) of 1.25 (1.03–1.52) and 1.22 (1.01–1.47), respectively. The mite sensitization rate was found to be the highest. Mite sensitization was associated with significant increases in the risks of AD, AR, and asthma, with ORs (95% CIs) of 2.15 (1.53–3.03), 1.94 (1.46–2.58), and 2.31 (1.63–3.29), respectively. Cockroach sensitization also increased the risk of asthma, with an OR (95% CI) of 2.38 (1.01–5.61). Mite sensitization was associated with carpet in the home and fungi on the house wall, and milk sensitization was associated with breastfeeding duration.

**Conclusion:**

Environmental exposures play a role in the development of allergic diseases. Allergen sensitizations were associated with certain environmental exposures. Early environmental interventions are urgently needed to prevent the development of childhood allergic diseases.

## Introduction

Over the past few decades, the prevalence of allergic diseases in children has rapidly increased in industrialized countries.^1^^,^^2^ In Taiwan, the prevalence of asthma may have increased dramatically,^3^ and asthma is the most common chronic illness among children.^4^ Most previous studies of atopic diseases conducted in Taiwan and abroad have investigated allergic asthma, which was found in about 38% of asthmatic patients.^5^ However, the incidence of atopic dermatitis (AD) in the developed world has also increased over the past several decades. AD is a chronic inflammatory skin disease with a peak onset in infancy, and a large majority of patients presenting skin symptoms in the first few years of life.^6^^,^^7^ In addition, allergic rhinitis (AR) is one of the most common chronic diseases in children.^8^ Because population genetic variability does not change with such rapidity, changing environmental factors are likely responsible for the increase in the number of individuals diagnosed with asthma, AD, and AR.^9^ Children with atopic diseases are most likely to have considerable school absences, family stress, and health care expenditures. Nowadays, most young children live in an indoor environment where many allergens exist. Therefore, the identification of indoor environmental factors among genetically susceptible children in Taiwan is urgently required.

Among environmental exposures, allergens are among the most important factors. Common indoor allergens for asthma include host dust mites, cockroaches, animal dander, and mold.^10^ Some studies have evaluated the environmental predictors of the early-life presentation of AD.^7^ For example, sensitization to food allergens (egg, milk, wheat, soy, and peanut) is associated with atopic dermatitis and is related to disease severity.^11^ Furthermore, other reported environmental factors, including cats, dogs, secondary tobacco smoke exposure, and air pollution, may trigger respiratory allergic diseases.^12^^,^^13^

Many studies of allergen sensitization in allergic asthma, AR, and AD have been conducted, although allergens and their associations with AD remain controversial. However, a large-scale environmental factors evaluation is lacking. Therefore, this study evaluated the relationships between environmental exposures, allergen sensitization, and allergic diseases in children. We conducted a cross-sectional study to investigate the prevalence of allergic diseases and allergen sensitization in a representative pediatric population in Taipei. We also investigated the associations among allergen sensitization of 6 chosen indoor allergens, environmental exposures, and the development of allergic diseases—asthma, AD, and AR.

## Methods

### Study population

We conducted a school-based survey for allergic diseases in kindergarten children in 2010. Schools were chosen through stratified systematic sampling in New Taipei City to ensure a degree of geographical and social diversity and provide a reasonably representative estimate. In each school, subjects were selected through cluster sampling. Children were recruited, and their written informed consent documents or those from their guardian were obtained. Parents were invited to complete a structured questionnaire. The exclusion criteria were inability to answer questions in Chinese, prematurity, and congenital and chronic diseases.

### Case definition

Asthma was defined as a positive response to physician-diagnosed asthma together with a positive response to nocturnal cough or exercise-induced wheezing in the past 12 months by using the International Study of Asthma and Allergies in Childhood (ISAAC) questionnaire. Cases of AD were defined using the questions “Has your child ever had AD diagnosed by a physician?” and” Has your child ever had a recurrent itchy rash for at least 6 consecutive half-month periods over the elbows, knees, face, wrists, neck, periauricular areas, or eyebrow areas?” Cases of AR were defined using the questions “Has your child ever been diagnosed as having AR by a physician?” and “Has your child ever had a problem with sneezing or a runny or blocked nose when your child did not have a cold or the flu?”

### Exposure measurements

The standard ISAAC-Chinese version questionnaire with the addition of questions concerning environmental allergen exposures were taken home by children and answered by their parents. Some information on basic demographic characteristics, residential environmental factors (such as environmental tobacco smoking, pets and cockroaches in the home, dampness of the house, fungus on the house wall, and carpets in the home), and family history of atopic diseases was also collected using the questionnaire.

### Skin prick test

Skin prick tests (SPTs) for 6 common allergens were performed, including house dust mites (HDMs mix, including *Dermatophagoides pteronyssinus* [Der p], *Dermatophagoides farinae* [Der f], *Dermatophagoides microceras* [Der m] and *Blomia tropicalis* [Blo t] allergens), dog dander, cockroaches, egg, milk, and crab allergens (ALK-Abelló, Round Rock, TX, USA). With a positive reaction to an allergen, the skin becomes itchy within a few minutes and then becomes red and swollen with a in the center. Histamine (0.1%) in phosphate buffered saline and normal saline were used as positive and negative controls, respectively. Children were advised to not take antihistamines for 72 h before the clinic appointment. The tests results were recorded in 15 min, and the mean wheal diameters were calculated (sum of the longest diameter and the diameter perpendicular to it divided by 2). In the presence of a positive control (>3 mm), a mean wheal diameter of at least 3 mm greater than the negative control was taken as positive for an allergen.

### Statistical analysis

Multiple logistic regressions were performed to estimate the association between environmental factors, allergen sensitization, and the development of allergic diseases, with adjustments made for potential confounders. The effect of environmental exposures on allergen sensitization was also evaluated. The confounders adjusted for in the model were selected based on the literature, and the standard statistical procedures 10% change in point estimate. All tests assumed a two-sided alternative hypothesis and a 0.05 significance level. All hypothesis tests were two-sided at the significance level of 0.05 and performed with SAS software version 9.1 (SAS Institute, Cary, NC, USA).

## Results

A total of 3192 children were recruited. Table 1 provides the demographic characteristics of the study population. Specifically, 485 (15.2%) children had AD, 1126 (35.3%) had AR, and 552 (17.3%) had asthma. Of all the risk factors collected, children with fungi on house walls had higher risks of AD [OR (95% CI): 1.27 (1.04–1.55)], AR [OR (95% CI): 1.18 (1.01–1.37)], and asthma [OR (95% CI): 1.22 (1.01–1.47)] (Table 2). Children with environmental tobacco smoke (ETS) exposure had higher risks of both AR and asthma, with ORs (95% CIs) of 1.23 (1.06–1.43) and 1.23 (1.06–1.52), respectively. Breastfeeding duration is the last risk factor noted. A breastfeeding duration of more than 6 months had a strong association with AD [OR (95% CI): 1.66 (1.20–4.31)].

**Table 1:** Demographic characteristics of participants

	All eligible participants (n = 3192)	
	n	%
***Children***		
**Infant gender** (%)		
Male	1725	54.1
Female	1467	45.9
**Premature birth** (<37 weeks) (%)^a^		
Yes	261	8.2
No	2833	88.8
Missing	98	3.0
**Birth body weight** (gm)		
Mean ± SD	3106.07 ± 457.961	
***Mother***		
**Maternal age at delivery** (years)		
Mean ± SD	29.32 ± 4.49	
**Maternal education** (%)		
Junior high school and below	185	5.8
Senior high school and above	3007	94.2
**Maternal Nationality** (%)		
Taiwan	3005	94.1
Foreign countries	187	5.9
**Maternal history of atopy** (%)		
Yes	1060	33.2
No	2132	66.8
**Family income per month** (NT$)∗ (%)		
< 600,000	930	29.2
≥600,000	2262	70.8

Abbreviations: SD, standard deviation; ∗NT$ per year.

a Number of participants does not add up to total N because of missing data

**Table 2:** Environmental risk factors for allergic diseases (N = 3192)

Characteristics	No. of Subject	AD Rate (%)	OR	95% CI	AR Rate (%)	OR	95% CI	Asthma Rate (%)	OR	95% CI
***Environment***										
**Duration of breast feeding** (%)										
No	669	11.4	1.00		29.6	1.00		10.8	1.00	
< 6 **months**	1577	15.2	1.39	(1.06–1.84)∗	32.7	1.16	(0.95–1.41)	13.0	1.24	(0.93–1.65)
≥ 6 **months**	535	17.6	1.66	(1.20–4.31)∗	27.7	0.91	(0.71–1.17)	14.4	1.39	(0.99–1.97)
**Older siblings** (%)										
No	866	16.1	1.00		37.6	1.00		16.9	1.00	
< 2	1631	15.7	0.97	(0.78–1.22)	35.1	0.90	(0.75–1.06)	16.7	0.99	(0.79–1.23)
≥ 2	563	13.5	0.82	(0.60–1.10)	32.7	0.80	(0.64–1.01)	18.1	1.09	(0.83–1.44)
**Day care** (%)										
No	2328	14.8	1.00		34.8	1.00		16.7	1.00	
Yes	704	17.8	1.25	(1.00–1.56)	35.8	1.05	(0.88–1.25)	17.0	1.03	(0.82–1.29)
**Furry pets at home** (%)										
No	2500	16.1	1.00		35.7	1.00		16.8	1.00	
Yes	550	13.6	0.82	(0.63–1.08)	33.6	0.91	(0.75–1.11)	17.3	1.03	(0.81–1.32)
**Cockroaches at home**										
No	557	14.9	1.00		34.5	1.00		14.7	1.00	
Yes	2541	15.3	1.03	(0.80–1.34)	35.5	1.07	(0.82–1.39)	17.6	1.23	(0.96–1.59)
**Carpets at home** (%)										
No	2947	15.0	1.00		32.3	1.00		16.8	1.00	
Yes	196	17.9	1.23	(0.84–1.80)	35.7	1.01	(0.75–1.37	21.9	1.40	(0.98–1.98)
**Fungi on house walls** (%)										
No	1873	14.4	1.00		33.8	1.00		15.8	1.00	
Yes	1168	17.6	1.27	(1.04–1.55)∗	37.6	1.18	(1.01–1.37)∗	18.6	1.22	(1.01–1.47)∗
**Environmental tobacco smoke exposure** (%)										
No	1282	15.9	1.00		32.4	1.00		15.2	1.00	
Yes	1758	15.0	0.93	(0.76–1.13)	37.1	1.23	(1.06–1.43)∗	18.3	1.25	(1.03–1.52)∗
**Incense burning at home** (%)										
No	1382	14.8	1.00		33.9	1.00		16.8	1.00	
Yes	1612	16.5	1.14	(0.94–1.39)	36.4	1.12	(0.96–1.30)	17.9	1.08	(0.90–1.31)
**Residence location**										
Urban	1009	17.1	1.00		47.4	1.00		18.4	1.00	
Country	38	7.9	0.41	(0.13–1.36)	35.5	0.61	(0.32–1.17)	15.6	0.82	(0.35–1.89)
**Living near main road**										
<1 km	326	14.4	1.00		35.0	1.00		17.8	1.00	
≥1 km	2524	15.7	1.11	(0.80–1.53)	37.7	0.89	(0.70–1.13)	17.0	0.95	(0.70–1.28)

∗P < 0.05.

Number of participants does not add up to total N because of missing data

Of all the allergens tested, the mite sensitization rate was found to be the highest, with mite sensitization found in 29% of all participants. The prevalence of other allergen sensitization by SPTs was lower than 4% for each one. The gender difference for allergen sensitization was not significant, except for mite; mite sensitization was slightly higher in boys (Table 3).

**Table 3:** The prevalence of allergen sensitizations based on skin prick tests

Characteristics	Total	Boys	%	Girls	%	P value
**House dust mite**	3192					0.007
–	2266	1191	69.0	1075	73.3	
**+**	926	535	31.0	391	26.7	
**Cockroach**						0.549
–	3092	1669	96.7	1423	97.1	
**+**	100	57	3.3	43	2.9	
**Dog dander**						0.926
–	3175	1717	99.5	1458	99.5	
**+**	17	9	0.5	8	0.5	
**Milk**						0.983
–	3166	1712	99.2	1454	99.2	
**+**	26	14	0.8	12	0.8	
**Egg**						0.366
–	3170	1712	99.2	1458	99.5	
**+**	22	14	0.8	8	0.5	
**Crab**						0.366
–	3170	1712	99.2	1458	99.5	
**+**	22	14	0.8	8	0.5	

The association between allergen sensitization and allergic diseases was significant for some aeroallergens. Mite sensitization was associated with significant increases in the risks of AD, AR, and asthma, with ORs (95% CIs) of 2.15 (1.53–3.03), 1.94 (1.46–2.58), and 2.31 (1.63–3.29), respectively. Cockroach sensitization also increased the risk of asthma, with an OR (95% CI) of 2.38 (1.01–5.61) (Table 4). However, the association of other aeroallergen and food allergen sensitization with allergic diseases failed to reach statistical significance after adjustments were made for confounding variables.

**Table 4:** Association of aeroallergen and food allergen sensitizations with allergic diseases

Skin prick test		Atopic dermatitis N = 485			Allergic rhinitis N = 1126			Asthma N = 552		
	Total N = 3192	n (%)	OR (95% CI)	Adjusted OR (95% CI)	n (%)	OR (95% CI)	Adjusted OR (95% CI)	n (%)	OR (95% CI)	Adjusted OR (95% CI)
***Aeroallergen*** Mite (−)	2266	277 (57.1)	1.00	1.00	669 (59.4)	1.00	1.00	309 (56.0)	1.00	1.00
(+)	926	208 (42.9)	2.08 (1.71–2.54)∗	2.15 (1.53–3.03)∗	457 (40.6)	2.33 (1.99–2.72)∗	1.94 (1.46–2.58)∗	243 (44.0)	2.25 (1.87–2.72)∗	2.31 (1.63–3.29)∗
Cockroach (−)	3092	466 (96.1)	1.00	1.00	1080 (95.9)	1.00	1.00	522 (94.6)	1.00	1.00
(+)	100	19 (3.9)	1.32 (0.79–2.20)	0.89 (0.30–2.63)	46 (4.1)	1.59 (1.06–2.37)∗	1.75 (0.79–3.85)	30 (5.4)	2.11 (1.36–3.27)∗	2.38 (1.01–5.61)∗
Animal (−) Dander	3175	481 (99.2)	1.00	1.00	1119 (99.4)	1.00	1.00	548 (99.3)	1.00	1.00
(+)	17	4 (0.8)	1.72 (0.56–5.31)	1.39 (0.15–12.65)	7 (0.6)	1.29 (0.49–3.39)	1.27 (0.21–7.76)	4 (0.7)	1.48 (0.48–4.54)	1.62 (0.18–14.80)
***Food allergen* Milk (−)**	3166	479 (98.8)	1.00	1.00	1110 (98.6)	1.00	1.00	543 (98.4)	1.00	1.00
**(+)**	26	6 (1.2)	1.68 (0.67–4.21)	2.41 (0.45–13.02)	16 (1.4)	2.96 (1.34–6.55)∗	1.17 (0.25–5.58)	9 (1.6)	2.56 (1.13–5.77)∗	2.06 (0.38–11.07)
**Egg (−)**	3170	479 (98.8)	1.00	1.00	1116 (99.1)	1.00	1.00	549 (99.5)	1.00	1.00
**(+)**	22	6 (1.2)	2.11 (0.82–5.41)	1.60 (0.16–15.65)	10 (0.9)	1.53 (0.66–3.56)	0.55 (0.06–5.40)	3 (0.5)	0.75 (0.22–2.56)	–
**Crab (−)**	3170	482 (99.4)	1.00	1.00	1116 (99.1)	1.00	1.00	548 (99.3)	1.00	1.00
**(+)**	22	3 (0.6)	0.88 (0.26–2.99)	0.98 (0.11–8.44)	10 (45.5)	1.53 (0.66–3.56)	0.36 (0.04–3.11)	4 (0.7)	1.06 (0.36–3.15)	–

Abbreviations: OR, odd ratio; CI, confidence interval; ∗P < 0.05.

#Model adjusted for gender, ETS exposure, fungi on house walls, and residence location

Mite sensitization was associated with carpets at home and fungi on the house wall, with ORs (95% CIs) of 1.44 (1.06–1.94) and 1.24 (1.06–1.46), respectively (Table 5). Cockroach sensitization was associated with cockroaches at home, and milk sensitization was associated with breastfeeding duration (Table 6). Nevertheless, no difference was observed in sensitization to animal dander and furry pets at home.

**Table 5:** Association of environment exposures with mite sensitizations

Characteristics	Total N = 3192	Mite sensitization		OR (95% CI)
		N	%	
Day care (%)				
No	2328	671	28.8	1.00
Yes	704	197	28.0	0.96 (0.80–1.16)
Furry pets at home (%)				
No	2500	731	29.2	1.00
Yes	550	151	27.5	0.92 (0.75–1.13)
Cockroaches at home				
No	557	165	29.6	1.00
Yes	2541	735	28.9	0.97 (0.79–1.18)
Carpets at home (%)				
No	2947	835	28.3	1.00
Yes	196	71	36.2	1.44 (1.06–1.94)∗
Fungi on house walls (%)				
No	1873	506	27.0	1.00
Yes	1168	368	31.5	1.24 (1.06–1.46)∗
Environmental tobacco smoke exposure (%)				
No	1758	505	28.7	1.00
Yes	1282	372	29.0	1.01 (0.87–1.19)
Incense burning at home (%)				
No	1382	387	28.0	1.00
Yes	1612	485	30.1	1.11 (0.94–1.30)
Residence location				
Urban	1009	274	27.2	1.65 (0.72–3.79)
Country	38	7	18.4	1.00
Living near main road				
<1 km	2524	735	29.1	0.96 (0.74–1.23)
≥1 km	326	98	30.1	1.00

∗P < 0.05.

Number of participants does not add up to total N because of missing data

**Table 6:** Association of environment exposures determined using a questionnaire with allergen sensitizations by skin prick tests

Environment exposures	Total N = 3192	Cockroach sensitization		OR (95% CI)
		N	%	
Cockroaches at home				
No	557	10	1.9	1.00
Yes	2541	88	3.5	1.96 (1.02–3.80)∗
		Animal dander sensitization		OR (95% CI)
		N	%	
Furry pets at home (%)				
No	2500	13	0.5	1.00
Yes	550	4	0.7	1.40 (0.46–4.32)
		**Milk sensitization**		OR (95% CI)
		N	%	
Duration of breast feeding (%)				
No	669	1	0.1	1.00
< 6 months	1577	12	0.8	5.12 (0.67–39.47)
≥ 6 months	535	10	1.9	12.72 (1.62–99.71)∗

∗P < 0.05.

Number of participants does not add up to total N because of missing data

## Discussion

This study demonstrated a fast-growing prevalence of allergic diseases in children in Taiwan. Participants with fungi on houses wall were found to have a higher risk of such diseases. Among the allergens selected, mite sensitization had the highest prevalence and was associated with significant increases in the risks of asthma, AR, and AD. Environmental tobacco smoke exposure, carpets at home, and breastfeeding duration were also important risk factors.

According to previous studies, childhood AD prevalence in Taiwan considerably increased from 7.2% in 1998 to 18% in 2002.^14^^,^^15^ In 2008, Ho et al revealed a prevalence of 10.7%.^16^ Our study found a prevalence of 15.2% in preschool children, which is compatible with the reported trend. The statistic is close to those reported in similar urbanization regions in Asia: 12%–13% in Japan^17^ and more than 11% in Korea.^18^ The prevalence of AR in industrialized countries has also dramatically increased to 20%–40% in children.^19^ It is consistent with our finding of 35.3%, which is higher than the previous Taiwan survey finding of 27.6% in 2002.^20^ For asthma, the present study revealed a prevalence of 17%, which is slightly higher than not only previous data from Taipei but also data from other industrialized countries.^21^^,^^22^ The allergic phenotype of asthma was estimated as 42%, which is also slightly higher than the previous literature. It may be attributed to differences in air pollution, humidity, lifestyle, temperature, and housing conditions.

The fast growing prevalence implies that environmental factors play a stronger role than genetics. Among all the risk factors collected in our study, children with ETS exposure and fungi on the house wall had higher risks of asthma and AR (Table 2). Our survey showed an increased risk of over 20% in children with ETS exposure, which is consistent with that in a previous meta-analysis.^23^ Another exposure, fungi on house walls, can be interpreted as visible mold and dampness. Our results are in line with those of previous European cohort studies,^24^^,^^25^ which demonstrated a higher risk in children exposed to any mold or dampness.

For AD, our study revealed significant odds ratios for breastfeeding duration and fungi on the house wall. Munblit D et al. claimed that conflicting evidence exists related to breastfeeding being a protector against allergic diseases among children.^26^ Based on the data, it may even be a risk factor for AD; however, we cannot exclude other potential benefits of breastfeeding; the association between breastfeeding and allergic disease development requires further investigation. In addition, there might exist a reverse causation when mothers whose children have AD tend to breastfeed their children.

Notably, in this study, the prevalence of mite sensitization was the highest among all allergens tested (Table 3). In addition to asthma and AR, our study showed that HDMs were crucial triggers of AD (Table 4). Because the allergy-related process of mites mainly occurs through the airway, it is widely accepted that the process predominantly influences asthma and AR. Whether HDM sensitization plays a role in the development of AD is still controversial. In a meta-analysis, Bremmer et al revealed no evidence of dust mite avoidance for AD prevention.^27^ However, the present study result supports the positive association between mite sensitization and AD. This may be explained by skin barrier destruction, thus rendering children vulnerable to contact with the allergen, or by systemic hypersensitivity to mites through IgE process. We highly suspect that there is a subgroup of potential children with AD who can benefit from mite avoidance; this requires further exploration.

Risk factors for specific allergen sensitization were identified in this study. We found that mite sensitization was associated with carpets in the home and fungi on house walls (Table 5). This result implies that an awareness of indoor humidity and routine carpet management are vital. Although we may not comprehensively remove all possible allergen particles, this would eliminate some important risk factors; measures for this include setting up a dehumidifier, simply removing any carpet-like furniture or other mite-avoidance procedures.^27^^,^^28^ Notably, only fungi on the house wall had a significant association with allergic diseases, not carpets in the home. Carpet-like furniture might be considered an allergens reservoir,^29^ which may enhance penetration of the causal pathway from mite exposure to mite sensitization and allergic diseases.

In the present study, we observed significantly higher HDM sensitization in boys than in girls (Table 3). Our study supports previous findings of a gender difference. Keller et al^30^ suggested that male participants have a higher risk of being allergic in childhood, perhaps due to anatomical differences and immune response profiles, which may cause boys to be more sensitive to allergens.

Furthermore, we demonstrated that only sensitization to particular allergens was associated with the same environmental exposures (Table 6). For example, cockroach sensitization was associated with cockroaches in the home. Because cockroaches may not appear when people are awake, a positive SPT finding may be indicative of a problem. Although no allergic symptom is noted yet, cockroaches at home are still a potential hazard and possibly induce allergic diseases, especially asthma.^31^ Another strong association noted in the study was that between milk sensitization and breastfeeding duration.

To investigate the associations among environmental exposures, allergen sensitization, and allergic disease development, we conducted SPTs as opposed to multiple allergen simultaneous tests (MASTs) through blood. Although it is noteworthy that a certain population had negative SPT results but had allergic symptoms or diseases, for kindergarten participants, SPT is preferred because it is painless, simple, and well tolerated. Furthermore, it is less expensive and more sensitive, and the results are available onsite within 20 min. We use SPTs as a convenient and noninvasive surrogate to evaluate the effect of whole environmental exposures on children in a population scale (Table 5). Our study showed that the SPT is informative and may serve as a convenient tool for detection of allergen sensitization after environmental exposures. This may help early prevention of the development of allergic diseases.

The present study has some potential limitations that may affect the interpretation of our results. First, we used a questionnaire for exposure evaluation without objective data. However, the validity of the questionnaire for exposure measurement, such as ETS and pet exposure, has been recognized, and it is an acceptable substitute for laboratory results.^32^^,^^33^ Moreover, some researchers have suggested that participants tend to underestimate exposures,^34^ which made the results toward the null. Thus, the questionnaire is still a suitable measure in such a large population. Secondly, some environmental risk factors were evaluated in an indirect way. However, it is very difficult to perform direct environmental exposure assessment in such a large-scale, population-based epidemiological study. Thus, SPTs were used as a convenient and noninvasive surrogate for allergen sensitization in a population scale, especially in children. We can still use the indirect evidences to inform a resident in the community through allergen sensitization profile that there might be some specific environmental risk factors for allergic diseases. Recall bias is another limitation, especially for respiratory outcomes. However, the recall of allergic disease status was assessed in a subgroup of the study population, and parental reports and medical records had favorable concordance. Finally, the cross-sectional study failed to demonstrate the causal relationship. Further follow-up is warranted.

Nevertheless, the study has several strengths. We performed the first population-based study demonstrating the relationships between allergic diseases, environmental risk factors, and allergen sensitization. This largest cross-sectional preschool survey in Taiwan included a considerable number of participants. No significant demographic difference was noted between the study cohort and the Taiwanese population of the same age. Our study also elaborated on comprehensive environmental factors with regards to potential health risks related to the pathway of the atopic process, which included both the skin and airways. These environmental exposures have common risk factors with great generalizability for further comparison with those in different countries. In addition, we administered an allergen sensitization survey through SPTs to identify many essential allergens. We discovered that both mite sensitization and fungi on house walls were significantly associated with all three common allergic diseases. Furthermore, mite sensitization had the highest prevalence. The results support the supposition that the avoidance of mites and house dampness might be beneficial for preventing the development of allergic diseases. Thus, our contribution can provide relevant information for patients in medical systems, for public education in communities and mass media, and for long-term health policy making.

## Conclusions

Environmental exposures play a role in the development of allergic diseases. Reduction in indoor allergen exposure and environmental risk factors may prevent the development of allergic diseases. Early environmental interventions are urgently needed.

## Availability of data and materials

The data that support the findings of this study are available on request from the corresponding author. The data are not publicly available due to their containing information that could compromise the privacy of research participants.

## Author contributions

C.-F.H. analyzed and interpreted the data, and wrote the manuscript. W.-C.C. interpreted the data and revised the manuscript. I.-J.W. collected, analyzed, and interpreted the data. I.-J.W. designed the research and wrote and revised the manuscript. All authors read and approved the final manuscript.

## Ethics approval

The study protocol was approved by the Institutional Review Board of Taipei Hospital, Ministry of Health and Welfare, Taiwan (TH-IRB-08-06), and it complied with the principles outlined in the Helsinki Declaration.

## Consent for publication

All the authors agree with the publication of this manuscript.

## Declaration of competing interest

The authors declare no competing interests.
